# Case report: Significant lesion reduction and neural structural changes following ibogaine treatments for multiple sclerosis

**DOI:** 10.3389/fimmu.2025.1535782

**Published:** 2025-02-06

**Authors:** David Qixiang Chen, José Adalberto Inzunza Domínguez, Juan Manuel Valle Uzeta, Abhiram P. Pushparaj, Jonathan E. Dickinson

**Affiliations:** ^1^ Ambio Life Sciences, Vancouver, BC, Canada; ^2^ Medical Department, Ambio Life Sciences, Vancouver, BC, Canada; ^3^ Consulting Department, +ROI Regulatory Advisory, Toronto, ON, Canada

**Keywords:** ibogaine, noribogaine, multiple sclerosis, psychedelic medicine, neuroregeneration, neuroimaging

## Abstract

Multiple sclerosis (MS) is a debilitating neurodegenerative disease characterized by demyelination and neuronal loss. Traditional therapies often fail to halt disease progression or reverse neurological deficits. Ibogaine, a psychoactive alkaloid, has been proposed as a potential neuroregenerative agent due to its multifaceted pharmacological profile. We present two case studies of MS patients who underwent a novel ibogaine treatment, highlighting significant neuroimaging changes and clinical improvements. Patient A demonstrated substantial lesion shrinkage and decreased Apparent Diffusion Coefficient (ADC) values, suggesting remyelination and reduced inflammation. Both patients exhibited cortical and subcortical alterations, particularly in regions associated with pain and emotional processing. These findings suggest that ibogaine may promote neuroplasticity and modulate neurocircuitry involved in MS pathology.

## 1 Introduction

Ibogaine is a naturally occurring indole alkaloid with complex neuropharmacology and strong oneirogenic (“waking dream generating”) properties. Although most widely discussed as an aid to mitigating withdrawal and cravings from opioids and other drugs (1–3), ibogaine has recently garnered attention for its potential to alleviate symptoms associated with traumatic brain injury (4), neuropathic pain (5), and other neurodegenerative conditions.

Ibogaine’s physiological effects appear to involve multiple mechanisms. Its interaction with N-methyl-D-aspartate (NMDA), σ2, and opioid receptors influence neural activity and plasticity (1, 6). Upregulation of both brain-derived neurotrophic factor (BDNF) and glial cell-derived neurotrophic factor (GNDF) promotes neuronal survival and plasticity (7–9). Reduction of pro-inflammatory cytokines decreases neuroinflammation (10). Improvements in cellular respiration may contribute to structural changes in neuronal cells (11, 12). Upregulation of myelination markers 2′, 3′-cyclic nucleotide 3′-phosphodiesterase (CNP) and myelin basic protein (MBP) mRNA demonstrate remyelination potential (13).

Multiple sclerosis (MS) is a chronic demyelinating disease characterized by neuroinflammation, axonal damage, and progressive neurological deficits (14). Standard treatments aim to modulate immune responses and slow disease progression but often have limited efficacy and significant side effects (15).

MS diagnosis relies on a combination of clinical evaluations and imaging studies. Cortical thickness analysis and diffusion-weighted imaging (DWI) are valuable neuroimaging tools for studying MS-related brain changes. Cortical thickness changes may reflect alterations to neurocircuitry, while DWI measures water diffusion to assess tissue integrity. Increased Apparent Diffusion Coefficient (ADC) values may suggest demyelination and axonal damage, whereas decreased ADC values may indicate improved neural integrity and reduced inflammation (16–22).

The present case report demonstrates the treatment effects of ibogaine on two patients who initially sought ibogaine for other reasons, but also had pre-existing diagnoses of MS. To our knowledge, this is the first documented case report suggesting neuroregenerative effects of ibogaine in human MS patients.

### 1.1 Case presentation

#### 1.1.1 Patient A

In February, 2023, an Ambio LIfe Science (“Ambio”) ibogaine treatment facility in Tijuana, Mexico received a 41-year-old male special forces veteran (“PA”) who was diagnosed with relapsing-remitting MS (RRMS), post-traumatic stress disorder (PTSD), major depressive disorder (MDD), and a traumatic brain injury (TBI) sustained ten years prior. He attended our program once previously, in September, 2022, at which time he suffered from vertigo believed to result from high alcohol intake. After his first treatment, vertigo symptoms resolved in the absence of alcohol consumption, only to return 2 months later, leading to his RRMS diagnosis.

**Figure 1:**
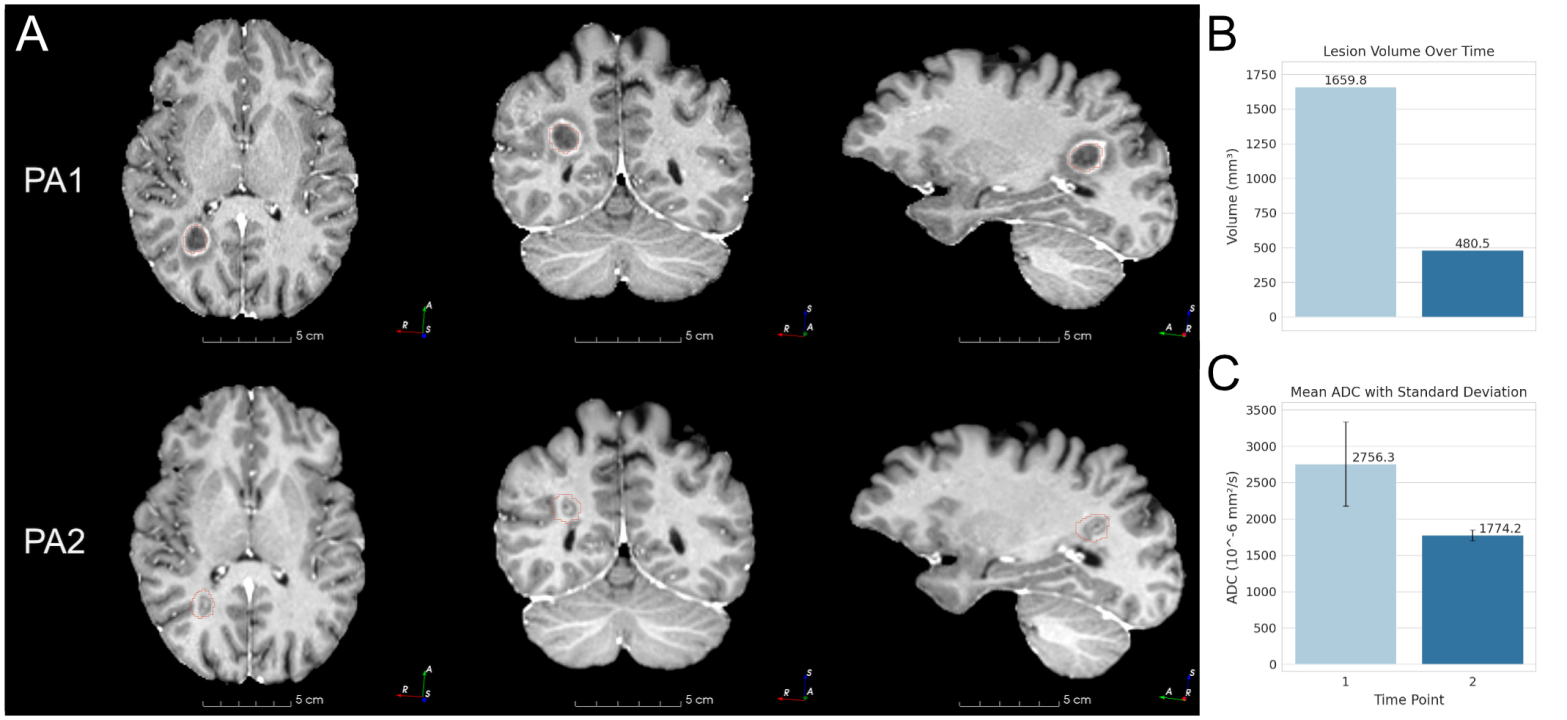
Patient A MRIs and lesion changes. **(A)** Patient A (PA) lesion MRI at each time point. PA1 is at 1 month, PA2 is progression at 3 months. The outline of the PA1 lesion segmentation mask is shown in red. The same PA1 mask is overlaid on PA2 for reference. **(B)** Lesion volumes at 1 month and 3 months. **(C)** Lesion mean ADC at the same time interval.

At intake, PA’s symptoms included progressive mobility impairment, coordination difficulties, some bladder control issues, bradypsychia, short-term memory deficits, and strong vertigo sensations every five to ten minutes. Emotional stress from relationship dissatisfaction was an additional factor influencing his overall health. His primary care physician prescribed dimethyl fumarate and Vitamin D to manage MS symptoms one month prior, but progressive neurological decline continued. Magnetic resonance imaging (MRI) revealed a large lesion in the right posterior parietal lobe, affecting cortical and subcortical white matter near the intraparietal sulcus (**Figure 1**).

#### 1.1.2 Patient B

Also in February 2023, Ambio received a 44-year-old female (“PB”) who was diagnosed with secondary progressive MS (SPMS) in 2018, as well as complex PTSD (CPTSD) from childhood trauma and a recent divorce following 17 years of marriage. Upon arrival, she exhibited severe muscle spasticity, hypotrophic extremities, difficulties with bladder and bowel control, and strong but infrequent attacks of vertigo. Although she could transfer herself using wall-mounted arm supports, she required a wheelchair for most activities. As of 4 months prior to intake, she was diagnosed “exercise intolerant”, precluding physiotherapy for more than 5 to 10 minute increments.

PB became confined to her mobility system after the installation of an intrathecal baclofen pump in 2019, which reduced the painful muscle spasticity that had previously allowed her to walk. She had taken glatiramer, high doses of Vitamin D, and also consumed cannabis on a regular basis to control muscle spasticity since 2018. Despite short-term success with ketamine therapy to try to control chronic pain, she terminated treatment infusions one year prior.

## 2 Methodology

### 2.1 Clinical protocol

Ibogaine is known to block the hERG potassium channel and prolong the cardiac QT interval, potentiating *torsades de pointes* and historically resulting in several cases of mortality and morbidity (23–26).

To avoid complications and improve treatment outcome, both patients underwent a novel ibogaine treatment protocol at Ambio, preceded by a physical intake, full metabolic panel, and electrocardiogram. 24-hour medical staff collected vitals every 30 minutes during waking hours. A patent-pending co-therapy protocol (27) was administered including: pre-treatment magnesium to prevent ibogaine-induced cardiac arrhythmia, vitamin infusions to promote cellular function, a “flood dose”, or “loading dose” of ibogaine hydrochloride (PA: 1200mg; PB: 500mg), followed by post-treatment lactulose to accelerate ibogaine metabolism. The ibogaine dosage was administered in 4 capsules over a 1.5-hour period. For the first 12 hours following the preliminary dosage, patients remained under constant cardiac monitoring to screen for arrhythmias including bradycardia. PB only accepted 2 of the 4 capsules, decreasing her overall dosage to accommodate an acute increase in her existing muscle spasticity. Once discharged, both patients continued a “microdose”, or “maintenance dosing” regimen (20mg/day).

The Multiple Sclerosis Quality of Life Index (MSQLI) and Hauser Ambulation Index (HAI) were used to quantify subjective changes and clinical observations.

### 2.2 Imaging analysis

DWI and anatomical structural MRI were acquired for each patient within two months prior to arrival and three (PA) to ten (PB) months after treatment. These images were used to generate cortical thickness measurements and ADC maps.

#### 2.2.1 DWI preprocessing

DWI data underwent initial preprocessing steps. Brain extraction was performed on the trace image using BET2 (Brain Extraction Tool) of the FMRIB software library [FSL; (28)]. The resulting brain mask was applied to the calculated ADC image to create a brain-extracted ADC.

#### 2.2.2 Cortical parcellation and subcortical segmentation

Cortical surface reconstruction and cortical thickness measurements were performed using the recon-all-clinical pipeline from FreeSurfer, optimized for heterogeneous clinical MRI scans (29). The pipeline incorporates machine learning-based segmentation methods, such as SynthSeg (30, 31) and SynthSR (32), to enhance robustness and reliability. SynthSeg allows for segmentation of brain MRI scans of any contrast and resolution, making it suitable for comparative analysis of heterogeneous clinical brain scans. SynthSR uses super-resolution to convert anatomical scans of varying contrasts into T1-weighted images suitable for cortical segmentation. The Desikan-Killiany atlas (33) was employed for cortical parcellation.

#### 2.2.3 Lesion segmentation

Lesion segmentation and quantification was performed manually with the 3D Slicer software suite (34). The lesion was outlined on the T1 anatomical MR image at each slice (35) to create a detailed lesion segmentation mask as the measurement region of interest (ROI). The lesion ROI is then subsequently projected to its intra-subject DWI space for ADC quantification.

#### 2.2.4 Anatomical and DWI registration

To align the anatomical (T1 or T2) images with the DWI space, a multi-stage registration process was implemented using ANTs (Advanced Normalization Tools) (36). The process involved the following steps: 1. Intra-subject longitudinal Rigid registration in anatomical space, from intrasubject time 2 to time 1. Diffeomorphic registration (SyN algorithm) from the DWI space to the anatomical space for each time point (37, 38). The ROIs for both cortical and lesion segmentations were then projected from anatomical to DWI space for ADC quantification.

#### 2.2.5 Clustering analysis

A Gaussian Mixture Model was used to perform clustering analysis on inter-hemispheric cortical thickness percentage changes to identify patterns of regional alterations and possible changes in neurocircuitry.

## 3 Results

### 3.1 Clinical observations

#### 3.1.1 Patient A

One day after treatment, PA subjectively noted a resolution of MS symptoms, including motor and bladder issues. 2 months post-treatment, MSQLI fatigue subscores dropped 92%. Bladder control issues completely resolved. The physical components summary score increased 24%, and mental components summary score improved by 42% (**Table 1**). Despite previous challenges walking because of an inability to coordinate foot movement, PA later reported participation in a 200 mile ultramarathon. One year after this second treatment episode, he still had not experienced any remission of vertigo.

**Table 1:** MSQLI data table.

Measure	Scale	Subscales	Patient A					Patient B				
			Baseline		1-month	2-months	% change	Baseline		1-month	2-months	% change
Multiple Sclerosis Quality of Life Index (MSQLI)	SF-36	Health Transition Item	1	Ibogaine loading dose, followed by microsoing.	1	1	0.00%	1	Ibogaine loading dose, followed by microsoing.	1	1	0.00%
		Physical Functioning Scale (PF)	70		100	100	42.86%	0		0	5	100.00%
		Role-Physical Scale (RP)	0		50	100	100.00%	0		0	50	100.00%
		Bodily Pain Scale (BP)	84		74	100	19.05%	12		84	100	733.33%
		General Health Scale (GH)	60		70	75	25.00%	57		67	72	26.32%
		Vitality Scale (VT)	15		60	70	366.67%	20		10	25	25.00%
		Social Functioning Scale (SF)	25		112.5	100	300.00%	12.5		62.5	25	100.00%
		Role-Emotional Scale (RE)	33.3		100	100	200.30%	0		100	66.7	100.00%
		Mental Health Scale (MH)	60		72	84	40.00%	60		72	80	33.33%
		Physical Components Summary Scale (PCS)	42.7		48.3	56.3	31.85%	21.7		25	35.2	62.21%
		Mental Component Summary Scale (MCS)	31.6		54.7	54.6	72.78%	36.3		54.5	46.5	28.10%
	MFIS	MFIS Total Score	52		29	4	-92.31%	53		47	41	-22.64%
		Physical Subscale	25		13	0	-100.00%	31		25	20	-35.48%
		Cognitive Subscale	22		12	4	81.82%	17		16	14	-17.65%
		Psychosocial Subscale	5		4	0	-100.00%	5		6	7	40.00%
	PES	PES Total Score	6		9	6	0.00%	22		8	6	-72.73%
	BLCS	BLCS Total Score	4		0	0	-100.00%	5		3	3	-40.00%
	BWCS	BWCS Total Score	0		0	0	0.00%	6		5	2	-66.67%
	IVIS	IVIS Total Score	0		0	0	0.00%	2		0	0	-100.00%
	PDQ	PDQ Total Score	42		40	33	-21.43%	40		30	25	-37.50%
		Attention/Concentration Subscale	13		15	11	-15.38%	14		11	10	-28.57%
		Retrospective Memory Subscale	12		9	7	-41.67%	8		4	2	-75.00%
		Prospective Memory Subscale	8		8	9	12.50%	5		4	4	-20.00%
		Planning/Organization Subscale	9		8	6	-33.33%	13		11	9	-30.77%
	MHI	MHI-18 Total Score	66		84	90	36.36%	75		77	89	18.67%
		Anxiety Subscale	64		76	80	25.00%	48		68	80	66.67%
		Depression Subscale	60		80	80	33.33%	70		70	70	0.00%
		Behavior Control Subscale	65		90	90	38.46%	85		85	100	17.65%
		Positive Affect Subscale	25		45	70	180.00%	65		50	75	15.38%
	MSSS	MSSS Total Score	41.7		59.6	57.8	38.61%	22.7		19.5	31.3	37.89%
		Tangible Support Subscale (TAN)	87.5		93.8	100	14.29%	12.5		50	56.3	350.40%
		Emotional/Informational Support Subscale (EMI)	12.5		28.1	31.3	150.40%	28.1		28.1	18.8	-33.10%
		Affectionate Support Subscale (AFF)	66.7		91.7	83.3	24.89%	25		0	25	0.00%
		Positive Social Interaction Subscale (POS)	0		25	16.7	100.00%	25		0	25	0.00%
Hauser Ambulatory Index (HAI)			1		1	1	0.00%	8		7	7	-12.50%

For each patient, a timeline of subjective symptom measures from Baseline to 2-month follow-up.

Health Status (SF-36), higher scores indicate better health. Modified Fatigue Impact Scale (MFIS), higher scores indicate a greater impact of fatigue on a patient’s activities. MOS Pain Effects Scale (PES), Higher scores indicate a greater impact of pain on a patient’s mood and behavior. Bladder Control Scale (BLCS), higher scores indicate greater bladder control problems. Bowel Control Scale (BWCS), higher scores indicate greater bowel control problems. Impact of Visual Impairment Scale (IVIS), higher scores indicate a greater impact of visual problems on daily activities. Perceived Deficits Questionnaire (PDQ), higher scores indicate greater perceived cognitive impairment. Mental Health Inventory (MHI), higher scores indicate better mental health. Modified MOS Social Support Survey (MSSS), higher scores indicate greater perceived support.

#### 3.1.2 Patient B

Despite acute increases in muscle spasticity under ibogaine’s effects, PB reported reductions in spasticity post-treatment. HAI scores improved from 8 (“restricted to wheelchair”) at baseline, to 7 (“walking limited to several steps with bilateral support”) immediately upon discharge, a change sustained at month 2 follow-up. PB increased the duration of physiotherapy from 10 minutes to 1 hour, and reported continued gradual improvement 2 years later. MSQLI scores improved 73% for chronic pain, and 29% for fatigue. Bladder control score improved from 5 to 3, and bowel control score improved from 6 to 2. Physical components summary score improved 38%, while mental components summary improved 22% (**Table 1**).

### 3.2 Brain imaging

#### 3.2.1 Patient A

Post-treatment analysis revealed a 71% reduction in lesion volume (from 1,659.8 mm³ to 480.5 mm³) and a 35.6% decrease in mean ADC (from 2,756.3 × 10⁻⁶ mm²/s to 1,774.2 × 10⁻⁶ mm²/s), suggesting reduced extracellular diffusion (**Figure 1**).

**Figure 2:**
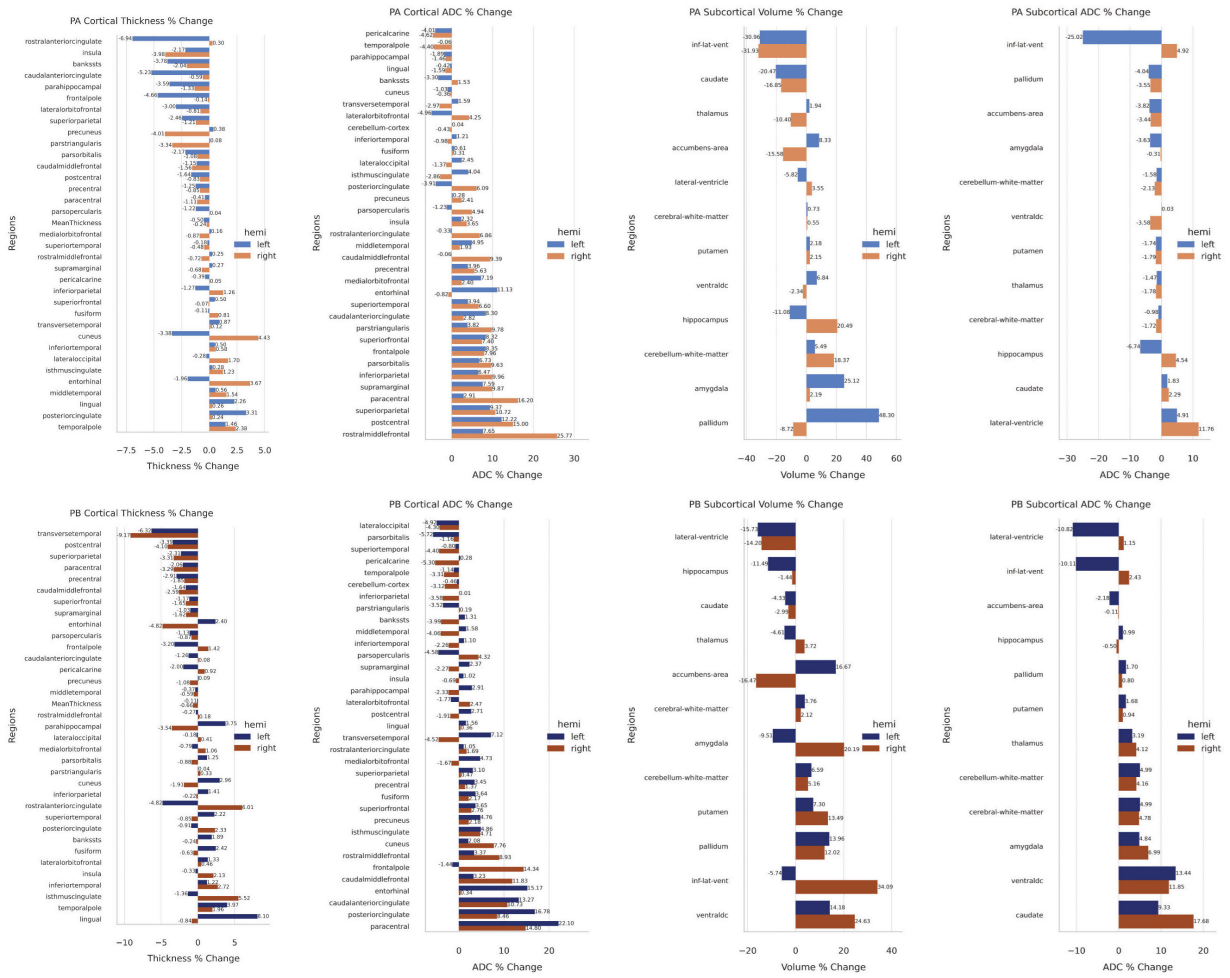
(Top) Patient A cortical and subcortical changes. (Bottom) Patient B cortical and subcortical changes.

Regions associated with emotional and cognitive processing exhibited cortical thinning, including the left rostral and caudal anterior cingulate cortices (ACC; 6.94% and 5.23%, respectively), left frontal pole (4.66%), and right precuneus (4.01%). In contrast, cortical thickening was noted in the right cuneus (4.43%) and entorhinal cortex (3.67%), with asymmetric decreases in the left cuneus (3.38%) and entorhinal cortex (1.96%) (**Figure 2**).

ADC changes varied across regions. Notable reductions were seen in the left lateral orbitofrontal cortex (4.96%) and right temporal pole (4.40%). Significant ADC increases included the right rostral middle frontal cortex (25.77%) and paracentral lobule (16.20%), and the left entorhinal cortex (11.13%) (**Figure 2**).

Ventricular volume decreased bilaterally (~31%), with asymmetrical ADC changes (left: -25.02%; right: +4.92%). The left pallidum (48.3%) and amygdala (25.12%) increased in volume, while hippocampal changes were asymmetric (right: +20.49%; left: -11.08%). These changes suggest hemispheric compensatory responses.

**Figure 3:**
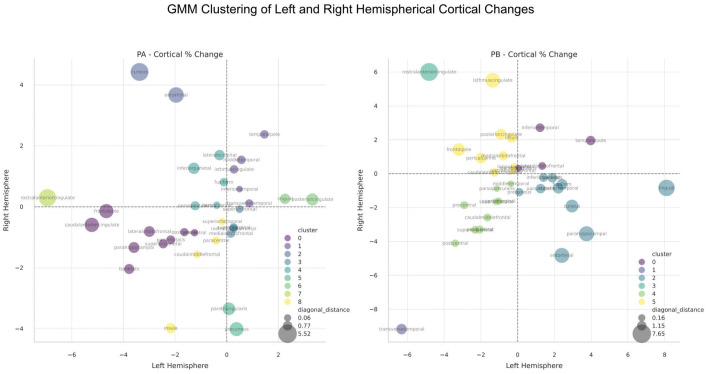
Gaussian Mixture Model (GMM) clustering analysis of cortical thickness changes between the hemispheres in Patient A (Left Panel) and Patient B (Right Panel). Clustering is based on the 4 quadrants of left/right changes (i.e both positive, both negative, etc.), as well as the distance from the diagonal, which represents the degree of regional change symmetry. The number of clusters were automatically determined by the GMM algorithm.

Gaussian Mixture Model analysis of cortical thickness changes identified three clusters (**Figure 3**). Regions associated with cortical thinning included areas involved in emotional processing, such as the ACC and insula. Regions showing symmetrical thickening or stability included areas related to memory and sensory integration, such as the middle temporal gyri and posterior cingulate cortex (PCC). Asymmetrical changes were observed in regions like the cuneus and entorhinal cortices, indicating differential hemispheric responses.

#### 3.2.2 Patient B

Cortical thinning was observed in several regions, including the right transverse temporal cortex (-9.17%) and left (-6.32%), and the right postcentral cortex (-4.10%) and left (-3.39%). Asymmetrical changes were notable in the entorhinal cortex, where the right decreased (-4.82%) and the left increased (+2.4%), and in the rostral ACC, where the left decreased (-4.82%) and the right increased (+6.01%). Conversely, cortical thickening was evident in regions such as the right isthmus cingulate (+5.52%) and left lingual cortex (+8.10%) (**Figure 2**).

ADC changes varied across regions. Decreases included the left pars orbitalis (-5.72%), right pericalcarine cortex (-5.30%), and left lateral occipital cortex (-4.92%). In contrast, ADC increases were more prominent, such as in the left paracentral lobule (+22.10%), right paracentral lobule (+14.8%), left (+16.78%), and right PCC (+8.46%). Other notable increases included the right frontal pole (+14.34%), left entorhinal cortex (+15.17%), and caudal ACC (left: +13.27%, right: +10.73%) (**Figure 2**).

Subcortical volume changes were also significant. The left and right lateral ventricle volumes decreased (-15.73% and -14.2%, respectively), while the right inferior lateral ventricle volume increased (+34.09%) as the left decreased (-5.74%). Asymmetrical changes were seen in the amygdala, with left volume decreasing (-9.51%) and right increasing (+20.19%), and in the accumbens area, with left volume increasing (+16.67%) and right decreasing (-16.47%). Increased volume was observed in the ventral diencephalon (right: +24.63%, left: +14.18%), pallidum (left: +13.96%, right: +12.02%), and putamen (left: +7.3%, right: +13.49%). Although caudate volume remained unchanged, ADC increased on both sides (left: +9.33%, right: +17.68%) (**Figure 2**).

Clustering analysis identified distinct patterns of cortical thickness changes (**Figure 3**). Regions with symmetrical thinning included clusters 1 and 4, encompassing areas associated with motor and executive functions, such as the paracentral lobules, postcentral gyrus, and transverse temporal cortex. These changes may reflect synaptic pruning and network optimization. Regions with symmetrical thickening, such as cluster 0, involved areas related to memory and executive functions, including the temporal pole and lateral orbitofrontal cortex. Asymmetrical left-side thickening and right-side thinning (cluster 2) were observed in memory and visual processing regions, including the entorhinal cortex, fusiform gyrus, and parahippocampal gyrus, possibly indicating neurogenesis and enhanced connectivity. Conversely, right-side thickening and left-side thinning (clusters 3 and 5) were prominent in regions associated with emotional regulation, including the anterior and isthmus cingulate cortices and medial orbital frontal cortex, suggesting differential hemispheric responses in emotional networks.

## 4 Discussion

Patients A and B both demonstrated significant cortical and subcortical neuroplastic changes following treatment, highlighting ibogaine’s potential in promoting adaptive neural remodeling. While the regions affected varied based on individual clinical presentations, the overarching neuroimaging patterns underscore ibogaine’s influence on pain, emotional regulation, cognitive processing, and motor function.

For Patient A (PA), a dramatic reduction in lesion volume and decreased ADC values may suggest possible remyelination and reduced extracellular space due to diminished inflammation or edema (20). These changes likely reflect improved cellular density, myelin integrity, and enhanced tissue function (17). PA also exhibited cortical thinning in the ACC and frontal pole, possibly associated with improved emotional regulation, as thinning in these areas may optimize neural circuitry through synaptic pruning (39). Conversely, cortical thickening in the PCC and temporal regions indicated potential neurogenesis or enhanced connectivity, which may support improved memory and sensory processing (40). Subcortical changes, such as decreased ADC in the hippocampus and amygdala, suggested enhanced tissue integrity, correlating with better emotional and memory processing. These findings aligned with PA’s significant improvements in mental health and cognitive function, emphasizing ibogaine’s impact on emotional and cognitive neurocircuitry.

Patient B (PB) similarly exhibited cortical and subcortical changes, with cortical thinning in motor regions likely reflecting synaptic pruning of maladaptive pathways, facilitating improved motor coordination. Cortical thickening in the entorhinal cortex and fusiform gyrus suggested enhanced neuroplasticity, potentially improving cognitive and visual processing. Subcortical ventricular alterations implied reduced neuroinflammation, contributing to symptom improvement. Clustering analysis revealed coordinated neuroplastic changes in motor and cognitive networks, aligning with PB’s marked reductions in pain and enhanced physical functioning.

Despite these differences, both patients shared common neuroplastic adaptations in emotional and affective neurocircuitry, particularly in regions such as the ACC, underscoring ibogaine’s role in modulating pain and emotional regulation networks (40). PA’s dramatic decrease in lesion volume and markers of inflammation were likely influenced by his diagnosis of RRMS, a less severe condition compared to PB’s SPMS, which may explain his greater degree of symptom remission and observed imaging improvements.

In both patients, clustering analyses highlighted that ibogaine facilitated coordinated changes across distinct neural networks, tailored to their individual pathologies. These findings suggest that ibogaine’s therapeutic effects are not only wide-ranging but also individualized, supporting its role in promoting adaptive neuroplasticity and clinical recovery. In this regard, it must be noted that the effects of ibogaine cannot be confirmed to be specific to MS and may have impacted the other neuropsychiatric comorbidities in these patients.

### 4.1 Possible mechanisms of regional changes

Remyelination is suggested by the lesion reduction and ADC decreases observed in Patient A, which may improve neural conductivity and reduce symptoms (41). Synaptic pruning may be responsible for the cortical thinning in specific regions, enhancing network efficiency and contributing to functional improvements (39). Neurogenesis or dendritic growth could be indicated by the cortical thickening and ADC increases, which may enhance connectivity and cognitive function. Additionally, decreased ADC in ventricles and subcortical areas may reflect reduced inflammation and edema, contributing to symptom relief.

While cortical thinning is generally linked to atrophy, it may also represent adaptive changes in the context of neuroplasticity. Synaptic pruning can enhance neural network efficiency, potentially leading to functional improvements despite reduced cortical thickness. This process is essential during development and may be recapitulated during neurorehabilitation.

### 4.2 Neurocircuitry involved

The modulation of pain pathways is suggested by changes in the ACC, insula, thalamus, and prefrontal cortex, which may contribute to reduced pain perception in both patients. Alterations in the cingulate cortex and frontal regions may enhance emotional processing, reducing symptoms of depression and anxiety. Remodeling in the precentral and postcentral gyri and paracentral lobules in Patient B may improve motor function and coordination. Furthermore, changes in the hippocampus, entorhinal cortex, and temporal regions may enhance memory processing and cognitive function.

Alterations in the PCC and ACC may suggest modulation of the default mode network (DMN). For PA, cortical thickening in the PCC may enhance DMN connectivity, improving cognitive functions like memory and self-awareness. For PB, changes in the PCC and ACC may alter DMN activity, reducing maladaptive rumination and pain perception. Modulation of the DMN may contribute to symptom relief in MS patients by improving functional connectivity and network efficiency.

## 5 Conclusion

These case studies suggest that ibogaine may induce neuroplastic and perhaps neuroregenerative changes in MS patients. The cortical and subcortical changes observed may represent adaptive processes contributing to clinical improvements. Modulation of the neurocircuitry related to pain and motor function may underlie these effects. Further research is needed to confirm these findings and explore ibogaine's therapeutic potential.

## Data Availability

The original contributions presented in the study are included in the article/**Supplementary Material**. Further inquiries can be directed to the corresponding author.

## References

[1] Alper KR. Ibogaine: a review. Alkaloids Chem Biol. (2001) 56:1–38. doi: 10.1016/S0099-9598(01)56005-8 11705103

[2] Brown TK. Ibogaine in the treatment of substance dependence. Curr Drug Abuse Rev. (2013) 6:3–16. doi: 10.2174/15672050113109990001 23627782

[3] Belgers M Leenaars M Homberg JR Ritskes-Hoitinga M Schellekens AF Hooijmans CR. Ibogaine and addiction in the animal model, a systematic review and meta-analysis. Transl Psychiatry. (2016) 6:e826. doi: 10.1038/tp.2016.71 27244235 PMC5545647

[4] Cherian KN Keynan JN Anker L Faerman A Brown RE Shamma A. Magnesium–ibogaine therapy in veterans with traumatic brain injuries. Nat Med. (2024) 30:373–81. doi: 10.1038/s41591-023-02705-w PMC10878970 38182784

[5] Dickinson JE Inzunza JAD Perez-Villa L Millar TG Pushparaj AP. Case report: Ibogaine reduced severe neuropathic pain associated with a case of brachial plexus nerve root avulsion. Front Pain Res. (2023) 4:1256396. doi: 10.3389/fpain.2023.1256396 PMC10502345 37720911

[6] Koenig X Hilber K. The anti-addiction drug ibogaine and the heart: a delicate relation. Molecules. (2015) 20:2208–28. doi: 10.3390/molecules20022208 PMC4382526 25642835

[7] He DY McGough NN Ravindranathan A Jeanblanc J Logrip ML Phamluong K. Glial cell line-derived neurotrophic factor mediates the desirable actions of the anti-addiction drug ibogaine against alcohol consumption. J Neurosci. (2005) 25:619–28. doi: 10.1523/JNEUROSCI.3959-04.2005 PMC1193648 15659598

[8] Carnicella S Kharazia V Jeanblanc J Janak PH Ron D. GDNF is a fast-acting potent inhibitor of alcohol consumption and relapse. Proc Natl Acad Sci. (2008) 105:8114–9. doi: 10.1073/pnas.0711755105 PMC2423415 18541917

[9] Marton S González B Rodríguez-Bottero S Miquel E Martínez-Palma L Pazos M. Ibogaine administration modifies GDNF and BDNF expression in brain regions involved in mesocorticolimbic and nigral dopaminergic circuits. Front Pharmacol. (2019) 10:193. doi: 10.3389/fphar.2019.00193 30890941 PMC6411846

[10] Tang BQ Li ZW Li L Li BJ Bian YQ Yu GD. New iboga-type alkaloids from Ervatamia officinalis and their anti-inflammatory activity. Fitoterapia. (2022) 156:105085. doi: 10.1016/j.fitote.2021.105085 34793883

[11] Paskulin R Jamnik P Zivin M Raspor P Strukelj B. Ibogaine affects brain energy metabolism. Eur J Pharmacol. (2006) 552:11–4. doi: 10.1016/j.ejphar.2006.09.008 17054944

[12] Paškulin R Jamnik P Danevčič T Koželj G Krašovec R Krstić-Milošević D. Metabolic plasticity and the energy economizing effect of ibogaine, the principal alkaloid of Tabernanthe iboga. J Ethnopharmacology. (2012) 143:319–24. doi: 10.1016/j.jep.2012.06.039 22751004

[13] Govender D, Moloko L Papathanasopoulos M Tumba N Owen G Calvey T. Ibogaine administration following repeated morphine administration upregulates myelination markers 2′, 3′-cyclic nucleotide 3′-phosphodiesterase (CNP) and myelin basic protein (MBP) mRNA and protein expression in the internal capsule of Sprague Dawley rats. Front Neurosci. (2024) 18:1378841. doi: 10.3389/fnins.2024.1378841 39114487 PMC11303312

[14] Dendrou CA Fugger L Friese MA. Immunopathology of multiple sclerosis. Nat Rev Immunol. (2015) 15:545–58. doi: 10.1038/nri3871 26250739

[15] Thompson AJ Banwell BL Barkhof F Carroll WM Coetzee T Comi G. Diagnosis of multiple sclerosis: 2017 revisions of the McDonald criteria. Lancet Neurol. (2018) 17:162–73. doi: 10.1016/S1474-4422(17)30470-2 29275977

[16] Fischl B. FreeSurfer. Neuroimage. (2012) 62:774–81. doi: 10.1016/j.neuroimage.2012.01.021 PMC3685476 22248573

[17] Hodaie M Chen DQ Quan J. Cortical thickness analysis in trigeminal neuralgia reflects unique changes related to treatment effect. Neuroimage. (2009) 47:S62. doi: 10.1016/S1053-8119(09)70301-5

[18] Calabrese M Reynolds R Magliozzi R Morra A Pitteri M. Cortical pathology and cognitive impairment in multiple sclerosis. Expert Rev Neurother. (2020) 20:831–45. doi: 10.1586/ern.10.155 21375447

[19] Filippi M Cercignani M Inglese M Horsfield MA Comi G. Diffusion tensor magnetic resonance imaging in multiple sclerosis. Neurology. (2001) 56:304–11. doi: 10.1212/WNL.56.3.304 11171893

[20] Chen DQ DeSouza DD Hayes DJ Davis KD O'Connor P Hodaie M. Diffusivity signatures characterize trigeminal neuralgia associated with multiple sclerosis. Multiple Sclerosis J. (2016) 22:51–63. doi: 10.1177/1352458515579440 25921052

[21] Zhong J Chen DQ Nantes JC Holmes SA Hodaie M Koski L. Combined structural and functional patterns discriminating upper limb motor disability in multiple sclerosis using multivariate approaches. Brain Imaging Behav. (2017) 11:754–68. doi: 10.1007/s11682-016-9551-4 27146291

[22] Laule C Moore GRW. Myelin water imaging to detect demyelination and remyelination and its validation in pathology. Brain Pathol. (2018) 28:750–64. doi: 10.1111/bpa.2018.28.issue-5 PMC8028667 30375119

[23] Thurner P Stary-Weinzinger A Gafar H Gawali VS Kudlacek O Zezula J. Mechanism of hERG channel block by the psychoactive indole alkaloid ibogaine. J Pharmacol Exp Ther. (2014) 348:346–58. doi: 10.1124/jpet.113.209643 24307198

[24] Alper K Bai R Liu N Fowler SJ Huang XP Priori SG. hERG blockade by iboga alkaloids. Cardiovasc toxicology. (2016) 16:14–22. doi: 10.1007/s12012-015-9311-5 25636206

[25] Alper KR Stajić M Gill JR. Fatalities temporally associated with the ingestion of ibogaine. J forensic Sci. (2012) 57:398–412. doi: 10.1111/j.1556-4029.2011.02008.x 22268458

[26] Mash DC. IUPHAR–invited review-Ibogaine–A legacy within the current renaissance of psychedelic therapy. Pharmacol Res. (2023) 190:106620. doi: 10.1016/j.phrs.2022.106620 36907284

[27] Inzunza J inventor; Ambio Life Sciences Inc, assignee. Ibogaine Treatment. World Intellectual Property Organization (2025). Available at: https://patentscope.wipo.int/search/en/detail.jsf?docId=WO2025000092 (Accessed January 28, 2025). WO2025000092.

[28] Smith SM. Fast robust automated brain extraction. Hum Brain Mapp. (2002) 17:143–55. doi: 10.1002/hbm.10062 PMC6871816 12391568

[29] Gopinath K Greve DN Das S Arnold S Magdamo C Iglesias JE. Cortical analysis of heterogeneous clinical brain MRI scans for large-scale neuroimaging studies. Medical Image Computing and Computer Assisted Intervention – MICCAI 2023. MICCAI 2023. Lecture Notes in Computer Science. Cham: Springer. (2023) 14227. doi: 10.1007/978-3-031-43993-3_4

[30] Billot B Greve DN Puonti O Thielscher A Van Leemput K Fischl B. SynthSeg: Segmentation of brain MRI scans of any contrast and resolution without retraining. Med Image Anal. (2023) 83:102789. doi: 10.1016/j.media.2023.102789 PMC10154424 36857946

[31] Billot B Magdamo C Arnold SE Das S Iglesias JE. Robust machine learning segmentation for large-scale analysis of heterogeneous clinical brain MRI datasets. Proc Natl Acad Sci. (2023) 120:e2216399120. doi: 10.1073/pnas.2216399120 36802420 PMC9992854

[32] Iglesias J Billot B Balbastre Y Magdamo C Arnold S Das S. SynthSR: a public AI tool to turn heterogeneous clinical brain scans into high-resolution T1-weighted images for 3D morphometry. Sci Adv. (2023) 9:eadd3607. doi: 10.1126/sciadv.add3607 36724222 PMC9891693

[33] Desikan RS Ségonne F Fischl B Quinn BT Dickerson BC Blacker D. An automated labeling system for subdividing the human cerebral cortex on MRI scans into gyral based regions of interest. Neuroimage. (2006) 31:968–80. doi: 10.1016/j.neuroimage.2006.01.021 16530430

[34] Fedorov A Beichel R Kalpathy-Cramer J Finet J Fillion-Robin J Pujol S. 3D Slicer as an image computing platform for the Quantitative Imaging Network. Magnetic resonance Imaging. (2012) 30:1323–41. doi: 10.1016/j.mri.2012.05.001 PMC3466397 22770690

[35] Chen DQ Quan J Guha A Tymianski M Mikulis D Hodaie M. Three-dimensional *in vivo* modeling of vestibular schwannomas and surrounding cranial nerves with diffusion imaging tractography. Neurosurgery. (2011) 68:1077–83. doi: 10.1227/NEU.0b013e31820c6cbe 21242825

[36] Avants BB Tustison NJ Song G Cook PA Klein A Gee JC. A reproducible evaluation of ANTs similarity metric performance in brain image registration. Neuroimage. (2011) 54:2033–44. doi: 10.1016/j.neuroimage.2010.09.025 PMC3065962 20851191

[37] Chen DQ Zhong J Hayes DJ Behan B Walker M Hung PS. Merged group tractography evaluation with selective automated group integrated tractography. Front Neuroanat. (2016) 10:96. doi: 10.3389/fnana.2016.00096 27790095 PMC5061742

[38] Chen DQ Dell’Acqua F Rokem A Garyfallidis E Hayes DJ Zhong J. Diffusion weighted image co-registration: investigation of best practices. BioRxiv. (2019) ):864108. doi: 10.1101/864108

[39] Vidal-Pineiro D Parker N Shin J French L Grydeland H Jackowski AP. Cellular correlates of cortical thinning throughout the lifespan. Sci Rep. (2020) 10:21803. doi: 10.1038/s41598-020-78471-3 33311571 PMC7732849

[40] Nevian T. The cingulate cortex: {{Divided}} in pain. Nat Neurosci. (2017) 20:1515–7. doi: 10.1038/nn.4664 29073647

[41] Murray PD McGavern DB Sathornsumetee S Rodriguez M. Spontaneous remyelination following extensive demyelination is associated with improved neurological function in a viral model of multiple sclerosis. Brain. (2001) 124:1403–16. doi: 10.1093/brain/124.7.1403 PMC5455988 11408335

