# Supplementary figures and images for: Case report: Significant lesion reduction and neural structural changes following ibogaine treatments for multiple sclerosis
Source: Front Immunol. 2025 Feb 6;16:1535782. doi: 10.3389/fimmu.2025.1535782 (PMC11839422; doi:10.3389/fimmu.2025.1535782)

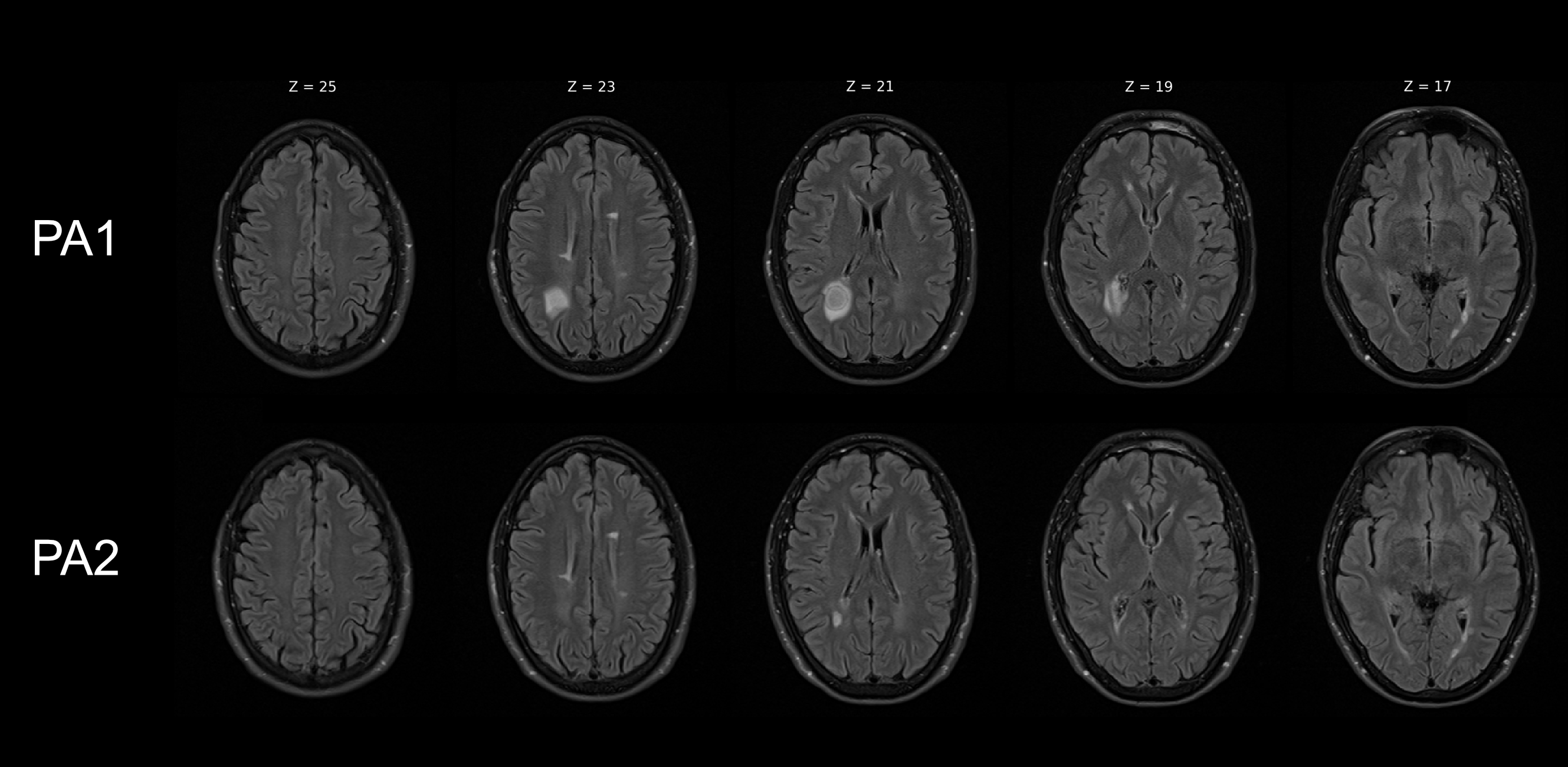

Supplement: Supplementary file 1 [file Image1.jpeg]

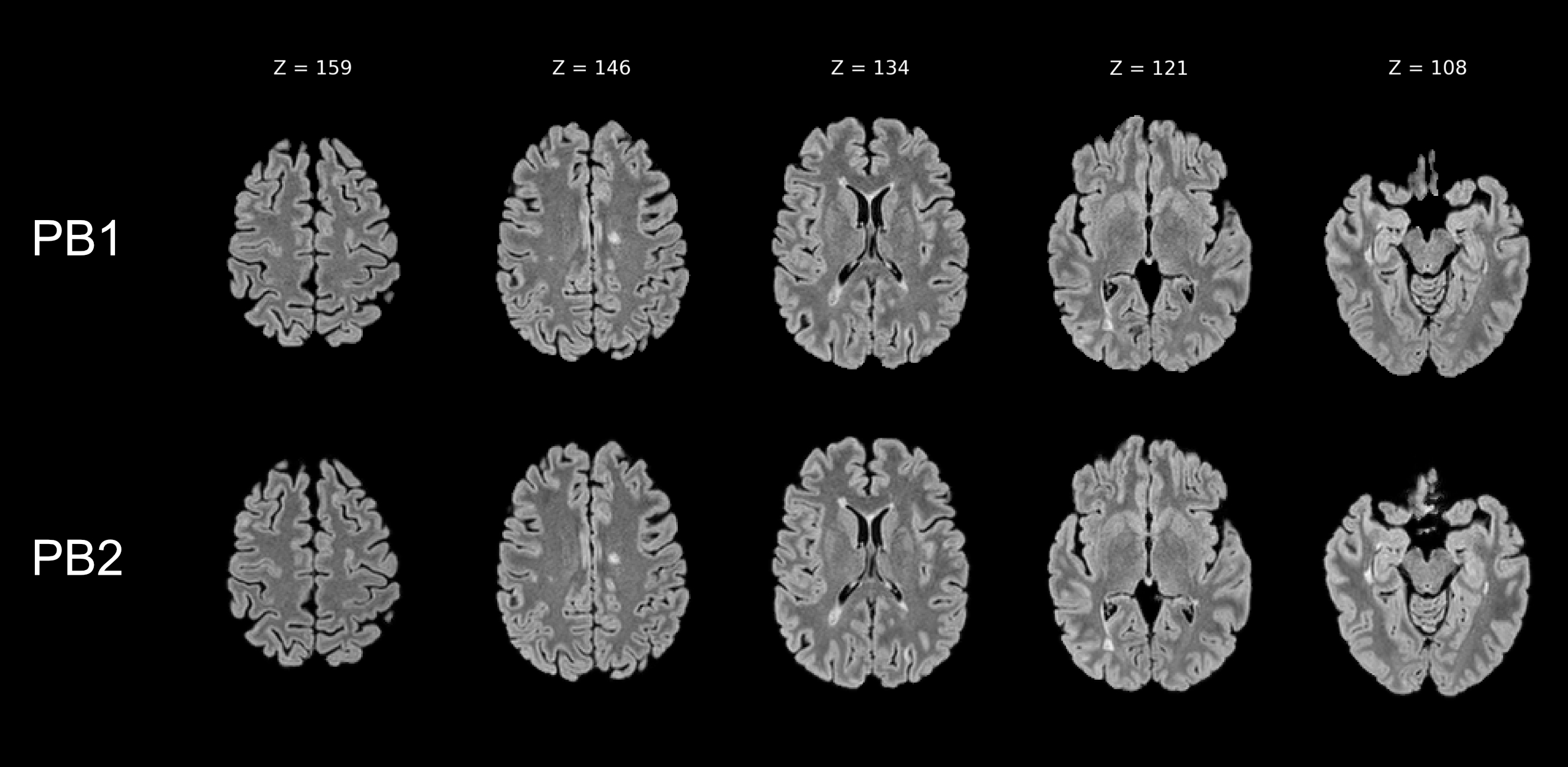

Supplement: Supplementary file 2 [file Image2.jpeg]
