# Supplementary material for: Case report: Significant lesion reduction and neural structural changes following ibogaine treatments for multiple sclerosis
Source: Front Immunol. 2025 Feb 6;16:1535782. doi: 10.3389/fimmu.2025.1535782 (PMC11839422; doi:10.3389/fimmu.2025.1535782)
Supplement: Supplementary file 3 [file DataSheet1.docx]

# ***Patient Perspectives***

#

# **Patient A**

#

# I can’t say with certainty when I developed Multiple Sclerosis. I was never athletic, and despite a strong desire in high school and college I never could develop my athletic talent enough to actually make it through try-outs and make the team in seemingly every sport I tried. I have no way of knowing if that was the beginning, or if I’m just inherently clumsy.

#

# I do know that 2 months after homecoming from my first deployment in Iraq in 2007 I developed a case of Optic Neuritis, which is frequently the first time people hear “multiple sclerosis” from a doctor. I was told it didn’t mean that I would develop MS, but it should definitely be on my radar. I brushed it off. I had failed in my attempt at SEAL training, and since I was an officer I couldn’t go back to try again. I was going to enlist in the National Guard’s Special Forces program and try to get into special operations via the path that remained open to me. I didn’t have space in my life for something like MS with a goal like that.

#

# I made it through the training and was assigned to a team after transitioning from the National Guard to active duty. Physically I did ok: I wasn’t the slowest, but I also was nowhere near being the fastest or the strongest, no matter how much work I put into it.

#

# In 2013 I received a Traumatic Brain Injury in Afghanistan from an IED. I began having more coordination issues and would frequently become dizzy. Emotional regulation went out the window. Again, it is hard to say where the MS began versus the legitimate symptoms of the TBI, but I feel like the MS was there long before that incident.

#

# I was selected to a special mission unit in a support role, having been dropped from the selection process after a year of extremely hard and dedicated training. Working at that level truly exposed the physical issues I had been able to hide when I was at 5^th^ Special Forces Group, and after a few years of desperately trying we decided that it was probably time for me to either move to a different role that wouldn’t be as physically demanding or go back to a Special Forces Group. I found a way to retire at that point by adding my 4 years at the Naval Academy to my total service time since I was now enlisted. I had terrible vertigo and lightheadedness and would frequently have to stop my car and collect myself while driving to and from work. I was terrified I would pass out at any moment. I was also drinking very heavily and assigned the blame for that dizziness and lightheadedness on being hungover nearly every day. MS didn’t even enter my mind as a possibility between the TBI, the drinking, severe PTSD, and a myriad of other issues. Without those complicating factors I think it may have been obvious at that point.

#

# I was introduced to Ibogaine through a study conducted by Stanford University in 2022 on its effects on Traumatic Brain Injury, which was my first experience at Ambio. I noticed significant improvement in my quality of life and returned from that experience much more functional in every way, and sober thanks to the Ibogaine eliminating my “need” to drink.

#

# In late October of that year, I began having terrible dizziness again and would wake up in the middle of the night holding on to the bed for dear life. My wife had to start driving me places. I was sober though, and so the return of these symptoms was a real concern: it wasn’t the daily hangover. I developed drop-foot in my left leg and could no longer run. I couldn’t sign my name with my left hand, which had largely stopped working and had no fine motor control.

#

# This led to my MS diagnosis in early January 2023. I began taking Tecfidera, but the side effects were so uncomfortable that I quickly decided that it was not a viable solution for me. Ambio invited me to come back for another Ibogaine treatment, optimistic that it may help ease the suffering.

#

# I stopped taking the Tecfidera and returned to Ambio in February and was absolutely amazed at what transpired. The day following the Ibogaine treatment I had full use of my body. I wrote page after page in my journal with my now-functional hand and wasn’t worried about falling when I walked. The dizziness was gone entirely. I microdosed Ibogaine for approximately 6 months, and my neurologist was shocked at how much my MRI’s cleared. The plaques on my brain no longer reacted to contrast and essentially no longer existed.

#

# In November of 2023 I completed a 10 mile swim and 200 mile run over 4 days to raise money for research into non-traditional PTSD treatments. To say that I had recovered would be an understatement.

#

# As of October 2024 I still am not on any traditional disease modifying treatments for my MS, nor do I feel any kind of need to be on any.

#

# Occasionally I’ll experience vertigo, numbness or pain. I get tired very quickly and am very sensitive to the heat and cold, so I can’t say that the MS was cured. But I can run 200 miles. I can live my life free to drive myself places. I’m not limited from participating in activities with my children. The gift that Ibogaine and Ambio have given me has changed my life and the lives of my family. I will forever be grateful for their kindness and willingness to try.

#

Sadly, Ibogaine is currently classified as a schedule I substance in the United States: no known legitimate medical use. I could not disagree with this description more. Ibogaine did what current pharmaceuticals could only dream of achieving, and in a single treatment. If I were to use Ibogaine in the United States it would make me a criminal. There are legitimate medical uses, and I am living proof of that. The laws of the United States are preventing people from having access to helping ease their suffering in an ethical manner with no side effects. I am fortunate to have been able to travel to Mexico to receive treatment, but thousands of people in my position don’t have that ability. This is a valid medical treatment that should be made available to all in need.

**Patient B**

I was 40 years old and already struggling with CPTSD when I was diagnosed with secondary progressive multiple sclerosis. I had been sick for many years, including the four years I helped care for my father, who died from ALS. I was devastated and frightened by my illness. My husband of almost 20 years left when I began to lose my ability to walk, very soon after my diagnosis. I found myself becoming severely disabled and living in my ex-husband's neighborhood, my children my only support and caregivers.

I was hopeless. My body had become so ravaged by MS. At the time, all four limbs and my core were violently stricken with severe spasticity. I was taking Percocet every two hours, even with a daily fentanyl patch. The pain and immobility of my limbs were unbearable. I was very close to being bed-bound. I could no longer drive and I was losing my ability to provide my own personal care.

In 2019, I had a surgery to relieve the severe spasticity in my legs by placing a medication pump into my spine. It greatly improved my leg spasticity and helped others care for me. It gave me just enough relief that I could really start to fight. I stopped using Percocet and fentanyl. Before the surgery, I couldn’t even bend my knees due to spasticity and pain. Now, they would bend, but they were very weak. Unfortunately, even with improvements I was continuing to rapidly decline in the area of muscle weakness. My leg muscles atrophied, significantly.

In 2020, I received my first dose of stem cells and I was following a high dose vitamin D protocol. I began working out for 5 to 10 minutes at a time, 5 to 10 times a day. I had a grab bar installed on my wall and I practiced squats, the only exercise I could do. Over time, I regained some muscle mass in my legs, but even though I arrived at a stable place in my MS diagnosis, I was still falling and needing help with basic life skills.

In an effort to become more independent, I began researching psychedelic medicine and brain healing. In 2022, I contacted Ambio to begin my journey, but my visit was postponed due to my inability to find support with travel. During this time of waiting, I began practicing breathwork, experienced a few ketamine treatments, started doing cold showers, and meditated and prayed every day to prepare for my ceremony. During my breathwork and cold therapy I felt myself slip into fits of rage, so much so that it frightened me. I always suspected that something happened to me when I was little. I grew up in a very unstable, unsafe family system.

When I finally traveled to Tijuana Mexico, I was greeted so warmly by the Ambio Life Science staff. I was loved and cared for immediately. I felt safe… finally. These kind, loving people became my family and I will always recall my memories of them when I need to feel love.

Ibogaine was not easy. Following the prompt of the psychologist, I prepared a question for this ancient plant medicine: Why am I so sick? Ibogaine answered immediately through my visions and my nervous system, drawing a direct connection between the emotional roots of my CPTSD, with all the stress it placed on my nervous system, and my MS.

Since my journey with Ibogaine, I have experienced profound healing and divine transformational insight. My spasticity and functionality in my whole body has greatly improved, as well as my strength. My cognitive abilities, including my spatial abilities and my executive function, have also greatly improved. I continue to work out daily, and these workouts have gone from 5 to 10 minutes to up to an hour at a time. I continue to work on my gait and leg strength, and I’ve gotten to the point where I can live independently.

More than anything, I’ve found hope that I can continue to improve over time. I finally worked up the courage to move back to my hometown, near my friends and family. And most importantly, my three boys are no longer a prisoner to my MS.

Ibogaine is an ancient, powerful, intense and loving therapy that needs your focus and interaction to experience. It touched emotional and physical aspects of my illness, and while it’s a divine healing, it simply unlocked the door. It’s given me a lot to work with, and provided improvement where nothing else did, but it’s taken time and persistence to achieve these gains.
