# Supplementary material for: Case report: Significant lesion reduction and neural structural changes following ibogaine treatments for multiple sclerosis
Source: Front Immunol. 2025 Feb 6;16:1535782. doi: 10.3389/fimmu.2025.1535782 (PMC11839422; doi:10.3389/fimmu.2025.1535782)
Supplement: Supplementary file 4 [file DataSheet2.docx]

***Supplementary Methods***

##

## **Imaging Protocol**

**Patient A**

The T1-weighted structural images were acquired using a 3D Magnetization Prepared Rapid Gradient Echo (MPRAGE) sequence on a 1.5T Siemens MAGNETOM Aera scanner. The acquisition employed a non-linear gradient correction and adaptive coil combination method using a 20-channel head and neck coil. The sequence parameters were as follows: TR = 2200 ms, TE = 2.98 ms, TI = 900 ms, flip angle = 15°, field of view = 100%, matrix size = 256 x 256, slice thickness = 1 mm, and in-plane phase-encoding direction = row. The images were obtained with GRAPPA parallel imaging (acceleration factor = 2) and 20% phase oversampling.

Diffusion-weighted images were acquired using a readout-segmented echo-planar imaging (rs-EPI) sequence, known as RESOLVE (REadout Segmentation Of Long Variable Echo-trains), on a 1.5T Siemens MAGNETOM Aera scanner. The images were obtained using a 20-channel head and neck coil, with adaptive coil combination. The sequence parameters were as follows: TR = 5700 ms, TE = 60.18 ms, flip angle = 180°, field of view = 100%, matrix size = 160 x 160, slice thickness = 5 mm, slice gap = 1.75 mm, and number of slices = 25. The diffusion-encoding scheme was monopolar, with a b-value of 1000 s/mm². Trace-weighted images were derived from the original diffusion-weighted data. GRAPPA parallel imaging (acceleration factor = 2) was employed. The effective echo spacing was 0.18 ms, with a total readout time of 28.62 ms. Fat suppression and 2D distortion correction were applied.

**Patient B**

3D FLAIR images were acquired on a 1.5T Siemens Aera MRI scanner. The images were acquired using a 20-channel head and neck coil with adaptive combine coil combination method. The sequence employed a T2-weighted turbo spin-echo (TSE) acquisition with variable flip angle (VFL) and inversion recovery preparation. Specific parameters included: TR = 5000 ms, TE = 335 ms, TI = 1600 ms, flip angle = 120°, and 1 mm isotropic resolution. The acquisition matrix was 256 x 220 x 192 (sagittal orientation), with 98% phase resolution and 87.5% phase field-of-view. GRAPPA parallel imaging was used with an acceleration factor of 2. The echo train length was 214, and the receiver bandwidth was 590 Hz/pixel. Fat suppression was applied.

Diffusion-weighted images were acquired on a 1.5T Siemens Aera MRI scanner using a single-shot echo-planar imaging (EPI) sequence. The images were obtained using a 20-channel head and neck coil with adaptive combine coil combination method. The protocol parameters were as follows: TR = 6300 ms, TE = 90 ms, flip angle = 90°, b-value = 1000 s/mm², and 3 mm slice thickness with 0.3 mm gap. The acquisition matrix was 128 x 128, reconstructed to 256 x 256, with a 100% field of view and 2 averages. Parallel imaging (GRAPPA) was employed with an acceleration factor of 2. The diffusion-encoding scheme was bipolar. Fat suppression was applied, and partial Fourier (factor 0.75) was used. The effective echo spacing was 0.39 ms, with a total readout time of 99.45 ms. Trace-weighted images were derived from the original diffusion-weighted data.

**ADC Map Calculation**

Apparent Diffusion Coefficient (ADC) maps were calculated using the following equation:

[
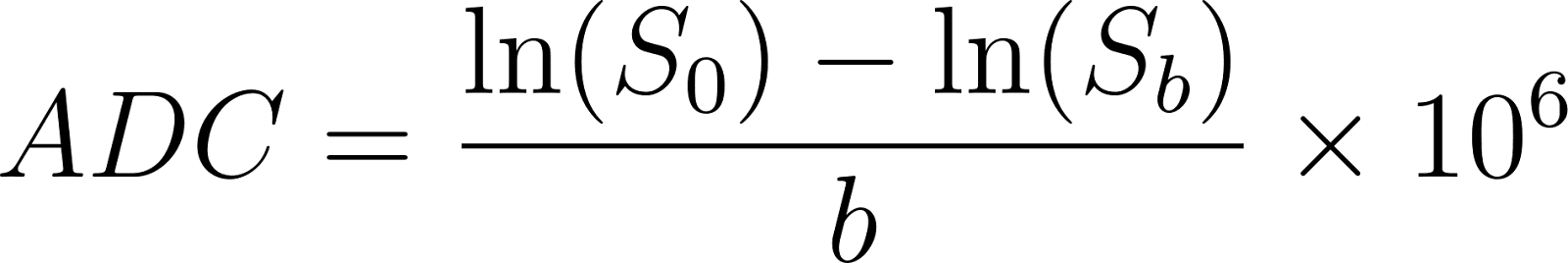
](https://www.codecogs.com/eqnedit.php?latex=%20ADC%20%3D%20%5Cfrac%7B%5Cln(S_0)%20-%20%5Cln(S_b)%7D%7Bb%7D%20%5Ctimes%2010%5E6%20#0)

Where [
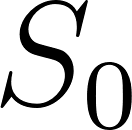
](https://www.codecogs.com/eqnedit.php?latex=S_0#0) is the signal intensity in the b0 image, [
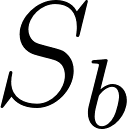
](https://www.codecogs.com/eqnedit.php?latex=S_b#0) is the signal intensity in the trace-weighted image, and *b* is the b-value used for the trace-weighted image. The resulting ADC values are expressed in units of [
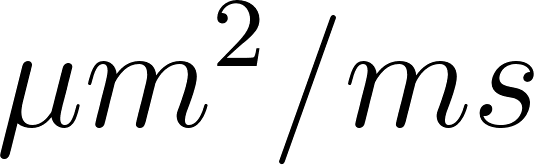
](https://www.codecogs.com/eqnedit.php?latex=%5Cmu%20m%5E2%2Fms#0).

### **Anatomical and DWI Registration**

To align the anatomical (T1 or T2) images with the DWI space, a multi-stage registration process was implemented using ANTs (Advanced Normalization Tools) \cite{avants2011reproducible}. The process involved the following steps: 1. Longitudinal Rigid registration in T1 space, from intrasubject time 2 to time 1. Diffeomorphic registration (SyN algorithm) from the DWI space to the T1 space within each subject for each time point.

For intra-subject anatomical alignment \cite{chen2016merged}, we employed a rigid transformation model with a gradient step length of 0.1, using Mutual Information as the similarity metric with 32 histogram bins. This registration used four resolution levels (shrink factors: 8, 4, 2, 1; smoothing sigmas: 3, 2, 1, 0 voxels) with 1000, 500, 250, and 100 iterations respectively, converging at a threshold of 1e-6 with a convergence window of 10.

For the DWI to anatomical space registration, we utilized a two-step approach \cite{chen2019diffusion}. First, an affine transformation was computed using Mutual Information as the similarity metric, with 32 histogram bins. This step employed a multi-resolution strategy with five levels (shrink factors: 5, 4, 3, 2, 1; smoothing sigmas: 4, 3, 2, 1, 0 mm), each running for 10,000 iterations. Subsequently, a deformable Symmetric Normalization (SyN) transformation was applied, using cross-correlation as the similarity metric with a 3x3x3 neighborhood. The SyN registration used three resolution levels (shrink factors: 3, 2, 1; smoothing sigmas: 2, 1, 0 mm) with 50, 35, and 15 iterations respectively. The gradient step size for the SyN transformation was set to 0.15 with a total of 3 time points.

All transformations were performed utilizing B-spline interpolation for structural images and nearest neighbor interpolation for region-of-interest (ROI) projections to preserve label integrity.
