# Supplementary material for: Case report: Significant lesion reduction and neural structural changes following ibogaine treatments for multiple sclerosis
Source: Front Immunol. 2025 Feb 6;16:1535782. doi: 10.3389/fimmu.2025.1535782 (PMC11839422; doi:10.3389/fimmu.2025.1535782)
Supplement: Supplementary file 5 [file DataSheet3.docx]

***GMM Clusters***

## **1 Patient A** Cluster 0: bankssts, caudalanteriorcingulate, frontalpole, lateralorbitofrontal, parahippocampal, parsorbitalis, postcentral, precentral, superiorparietal

Cluster 1:inferiortemporal, isthmuscingulate, middletemporal, temporalpole, transversetemporal

Cluster 2: cuneus, entorhinal

Cluster 3: medialorbitofrontal, rostralmiddlefrontal, superiorfrontal, supramarginal

Cluster 4: fusiform, inferiorparietal, lateraloccipital, parsopercularis, pericalcarine

Cluster 5: parstriangularis, precuneus

Cluster 6: lingual, posteriorcingulate

Cluster 7: rostralanteriorcingulate

Cluster 8: caudalmiddlefrontal, insula, paracentral, superiortemporal

**1.2 Patient B**

Cluster 0: inferiortemporal, lateralorbitofrontal, parstriangularis, temporalpole

Cluster 1: caudalmiddlefrontal, middletemporal, paracentral, parsopercularis, postcentral, precentral, superiorfrontal, superiorparietal, supramarginal, transversetemporal

Cluster 2: bankssts, cuneus, entorhinal, fusiform, inferiorparietal, lingual, parahippocampal, parsorbitalis, precuneus, superiortemporal

Cluster 3: rostralanteriorcingulate

Cluster 4: caudalanteriorcingulate, frontalpole, insula, isthmuscingulate, lateraloccipital, medialorbitofrontal, pericalcarine, posteriorcingulate, rostralmiddlefrontal
